# Regulation of Akt-mTOR, ubiquitin-proteasome and autophagy-lysosome pathways in locomotor and respiratory muscles during experimental sepsis in mice

**DOI:** 10.1038/s41598-017-11440-5

**Published:** 2017-09-07

**Authors:** Jérome Morel, Jean-Charles Palao, Josiane Castells, Marine Desgeorges, Thierry Busso, Serge Molliex, Vanessa Jahnke, Peggy Del Carmine, Julien Gondin, David Arnould, Anne Cécile Durieux, Damien Freyssenet

**Affiliations:** 1 Univ Lyon - University Jean Monnet Saint Etienne; Inter-university Laboratory of Human Movement, EA7424, F-42023 Saint Etienne, France; 2 Département d’anesthésie et réanimation, Centre Hospitalier Universitaire de Saint Etienne, Saint Etienne, France; 3 Institut NeuroMyoGène, Université Claude Bernard Lyon 1, INSERM U1217, CNRS UMR 5310 Villeurbanne, France

## Abstract

Sepsis induced loss of muscle mass and function contributes to promote physical inactivity and disability in patients. In this experimental study, mice were sacrificed 1, 4, or 7 days after cecal ligation and puncture (CLP) or sham surgery. When compared with diaphragm, locomotor muscles were more prone to sepsis-induced muscle mass loss. This could be attributed to a greater activation of ubiquitin-proteasome system and an increased myostatin expression. Thus, this study strongly suggests that the contractile activity pattern of diaphragm muscle confers resistance to atrophy compared to the locomotor *gastrocnemius* muscle. These data also suggest that a strategy aimed at preventing the activation of catabolic pathways and preserving spontaneous activity would be of interest for the treatment of patients with sepsis-induced neuromyopathy.

## Introduction

Intensive care unit (ICU)-acquired weakness is a frequent complication of critical illnesses associated with a high mortality and morbidity. ICU-acquired weakness affects both locomotor and respiratory skeletal muscles, begins early during the episode of critical illness, and contributes to exercise limitations and a reduced quality of life^1–3^ that can persist for months and years after hospital discharge^1^.

Weakness of peripheral limb muscles in ICU patients is due to a decrease in skeletal muscle mass and a reduction in force production. ICU patients receiving prolonged mechanical ventilation had marked decrements in grip strength, a marker for peripheral muscle strength and a poor hospital outcome^2^. More recently, Puthucheary *et al*. also reported a 20% decrease in the cross-sectional area of *rectus femoris* 10 days after admission of patients in ICU^4^. Respiratory muscles are also prone to deconditioning. In patients with sepsis, the main reason for ICU admission and a major risk factor for ICU-acquired weakness^5,6^, the volume of diaphragm muscle is decreased by 27%, 25 days after ICU admission^7^. At the histological level, the cross-sectional area of diaphragm muscle fibers is decreased by 50% in mechanically ventilated ICU patients^8^. Altogether, loss of mass and function of locomotor and respiratory muscles has been associated with a longer duration of mechanical ventilation, an increase in associated complications and higher hospital mortality^2,9,10^.

In mammals, muscle mass is tightly regulated by the complex interplay of critical signaling pathways, which are coordinately regulated and share common molecular partners. The IGF1-Akt pathway allows the coordinated regulation of both protein synthesis and protein degradation. Activation of the Akt-mTOR axis is critical for the activation of protein translation initiation^11^, whereas activation of the Akt-FoxO axis is critical for the repression of protein degradation by preventing the expression of E3-ubiquitin ligases and autophagy-related genes, involved in ubiquitin-proteasome proteolysis^12,13^ and autophagy-lysosome proteolysis^14,15^, respectively. Smad signaling pathways are also important regulators of skeletal muscle mass. Activation of the BMPs-Smad1/5/8 pathway limits the extent of skeletal muscle atrophy in response to fasting, denervation and cerebral ischemia by inhibiting the expression of the E3-ubiquitin ligases and activating the IGF1-Akt-mTOR axis^16–18^. Conversely, myostatin, a master negative regulator of skeletal muscle mass^19–21^, leads to the activation of Smad2 and Smad3, which together with the regulatory Smad4 inhibits the Akt-mTOR axis^20^, decreases muscle gene expression^21^ and stimulate proteolysis^22^.

Previous studies indicate that an increase in muscle protein degradation, and to a lesser extent a decrease in protein synthesis, are involved in the atrophy of locomotor muscles in patients with sepsis^4,23–25^ and in animal models of sepsis^26–30^. Myostatin expression in rat locomotor muscles is also transitory increased 4 days after the injection of zymosan, a glucopolysacharride that induces non-bacterial peritonitis, suggesting that myostatin is also a critical catabolic signal involved in skeletal muscle atrophy^31^. However, very little is known about the extent, time course and regulation of these pathways in skeletal muscle during sepsis. This is of clinical relevance since a prolonged unbalance between anabolic and catabolic pathways can lead to profound alterations in muscle function that may compromise the recovery of patients with sepsis. Furthermore, locomotor and respiratory muscles may not be similarly affected by sepsis. Although mechanical ventilation by itself may induce progressive dysfunction and atrophy of diaphragm muscle^8^, sepsis has also been reported as a worsen condition that increased diaphragm atrophy in mechanically ventilated patients^7^. Therefore, diaphragm atrophy may be also causally related to the infection disease *per se*. Whether sepsis similarly or differentially regulates anabolic and catabolic pathways in locomotor and respiratory muscles remains to determine.

The aim of this study was therefore to gain insight into the mechanisms underlying skeletal muscle loss in ICU patients with sepsis, by conducting a time course analysis in a mice model of sepsis without mechanical ventilation. We hypothesize that locomotor muscles are more sensitive to sepsis-induced muscle atrophy than respiratory muscles. The specific pattern of motor nerve activity in diaphragm muscle, which is obviously preserved during spontaneous ventilation, would limit the extent of muscle atrophy in response to sepsis. The comparative analysis of the molecular mechanisms triggered in locomotor and respiratory muscles in response to sepsis will provide information about the causative role of sepsis in respiratory muscle weakness.

## Results

CLP induced an acute peritonitis with symptoms of severe illness (hypophagia, lethargy) being present in all mice during the first 3 days after surgery. However, a number of animals died prematurely because of the severity of the infection. All animals that had survived 4 days after CLP remained alive for the rest of the experimental period. Therefore, the mortality varied greatly between animals, even though post-mortem examination of the peritoneal cavity did not show any visual differences in the size of peritonitis between animals. Differences in mortality may be more specifically related to the capacity of the mouse to face the infectious episode^32^. Overall, the survival rate was 41% one week after CLP (Supplementary Fig. 1).

### Sepsis induces atrophy of *gastrocnemius* muscle but not diaphragm muscle

**Figure 1:**
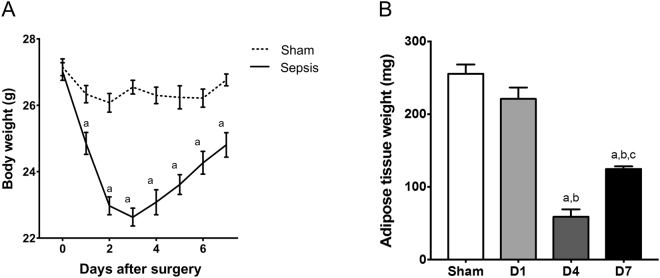
Effects of sepsis on body weight and adipose tissue weight. Body weight (n = 9–11/group) (**A**) and adipose tissue weight (n = 9–11/group) (**B**) in sham and sepsis mice. D1: one day after CLP; D4: 4 days after CLP; D7: 7 days after CLP. Data are expressed as means ± SEM. a: significantly different from Sham mice; b: significantly different from D1; c: significantly different from D4.

The initial body mass pre-insult was similar between sham and sepsis mice. The body weight response to sepsis allows clearly delineating two different phases, a catabolic phase that reached a nadir 3 days after CLP, and a progressive recovery phase from day 4 to day 7 (Fig. 1A), even though body weight had not returned to control values at the end of the experimental period. This biphasic behavior was also clearly evidenced by the changes in adipose tissue weight after CLP (Fig. 1B).

**Figure 2:**
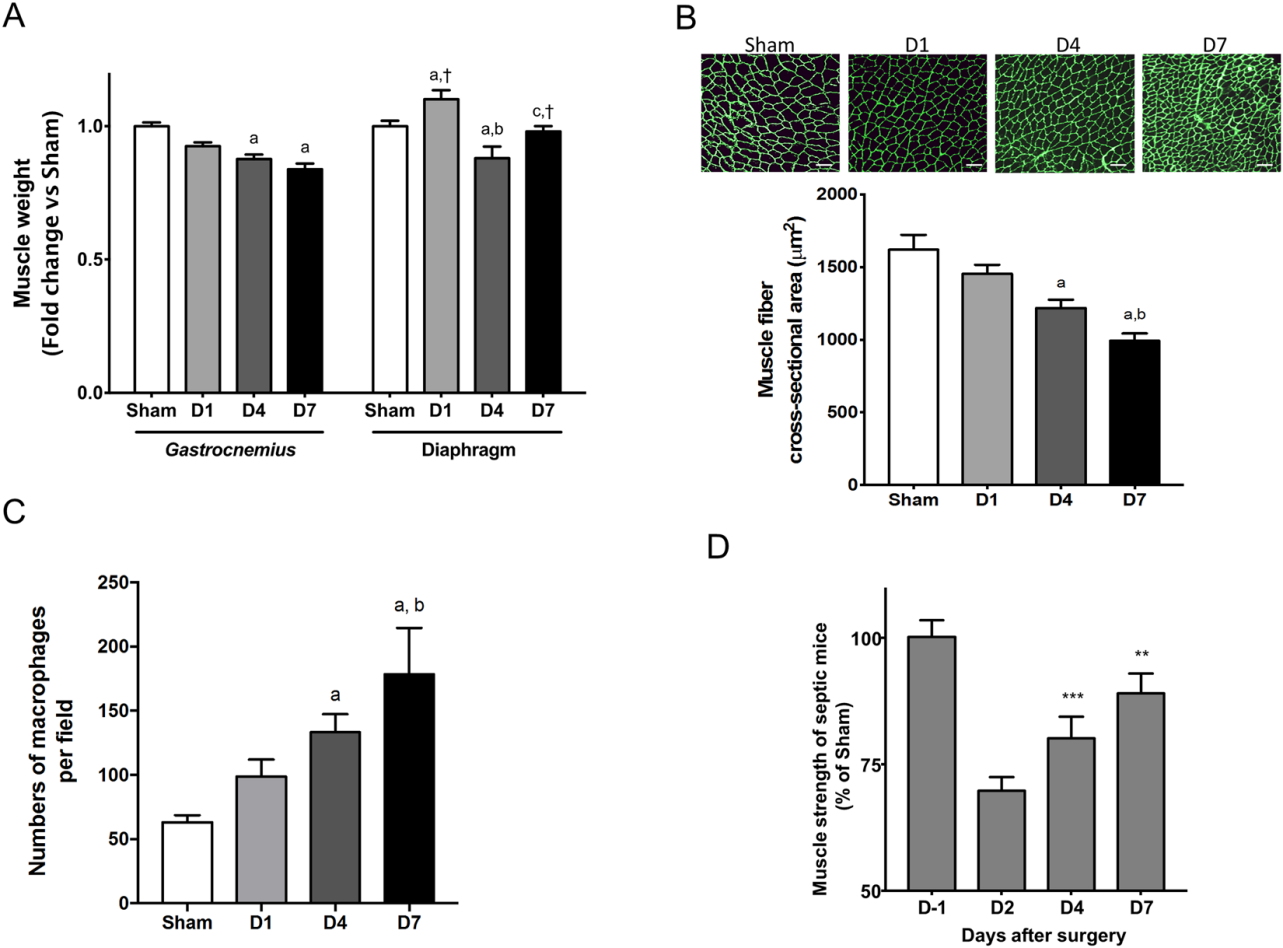
Sepsis induces atrophy of locomotor muscles but no respiratory muscle. *Gastrocnemius* (n = 9–11/group) and diaphragm (n = 9–11/group) muscle weights (**A**). Representative images of a cross-section of *gastrocnemius* muscle immunostained with laminin and quantification of muscle fiber cross-sectional area (n = 8–9/group) (**B**). Numbers of macrophages per field (n = 5–8/group) (**C**). Forelimb and hindlimb muscle force (n = 9/group) (**D**). D-1: one day before CLP; D1: one day after CLP; D2: 2 days after CLP; D4: 4 days after CLP; D7: 7 days after CLP. Data are expressed as means ± SEM. a: significantly different from Sham mice; b: significantly different from D1; c: significantly different from D4. †: significantly different from *gastrocnemius* muscle at the same time point. **p < 0.01 and ***p < 0.001: significantly different from D1.

One main feature of the present study was the differential response between locomotor *gastrocnemius* and respiratory diaphragm muscles. By contrast to the mass of diaphragm muscle, which remained unchanged 7 days after CLP, sepsis induced a progressive decrease in *gastrocnemius* muscle mass that reached 16%, 7 days after CLP (Fig. 2A). Accordingly, the cross-sectional area of *gastrocnemius* muscle fibers progressively decreased during the time course of the experiment (Fig. 2B). Immunofluorescence staining of F4/80 was performed to determine the presence of macrophages in the *gastrocnemius* muscle (Fig. 2C). Macrophage content was significantly higher at D4 (2.1-fold) and at D7 (2.8-fold) for CLP mice as compared to Sham. A significant increase between D1 and D7 was also observed.

Loss of skeletal muscle mass was also observed in *extensor digitorum longus, quadriceps*, *soleus* and *tibialis anterior* muscles (Supplementary Fig. 2). Not surprisingly and in agreement with the data above, loss of skeletal muscle mass was paralleled by a pronounced decrease in muscle strength 2 days after CLP (Fig. 2D). Muscle force then progressively recovered, without reaching the control values at day 7.

### Sepsis increases phosphorylation of rpS6 and p70^S6k^ in *gastrocnemius* muscle

**Figure 3:**
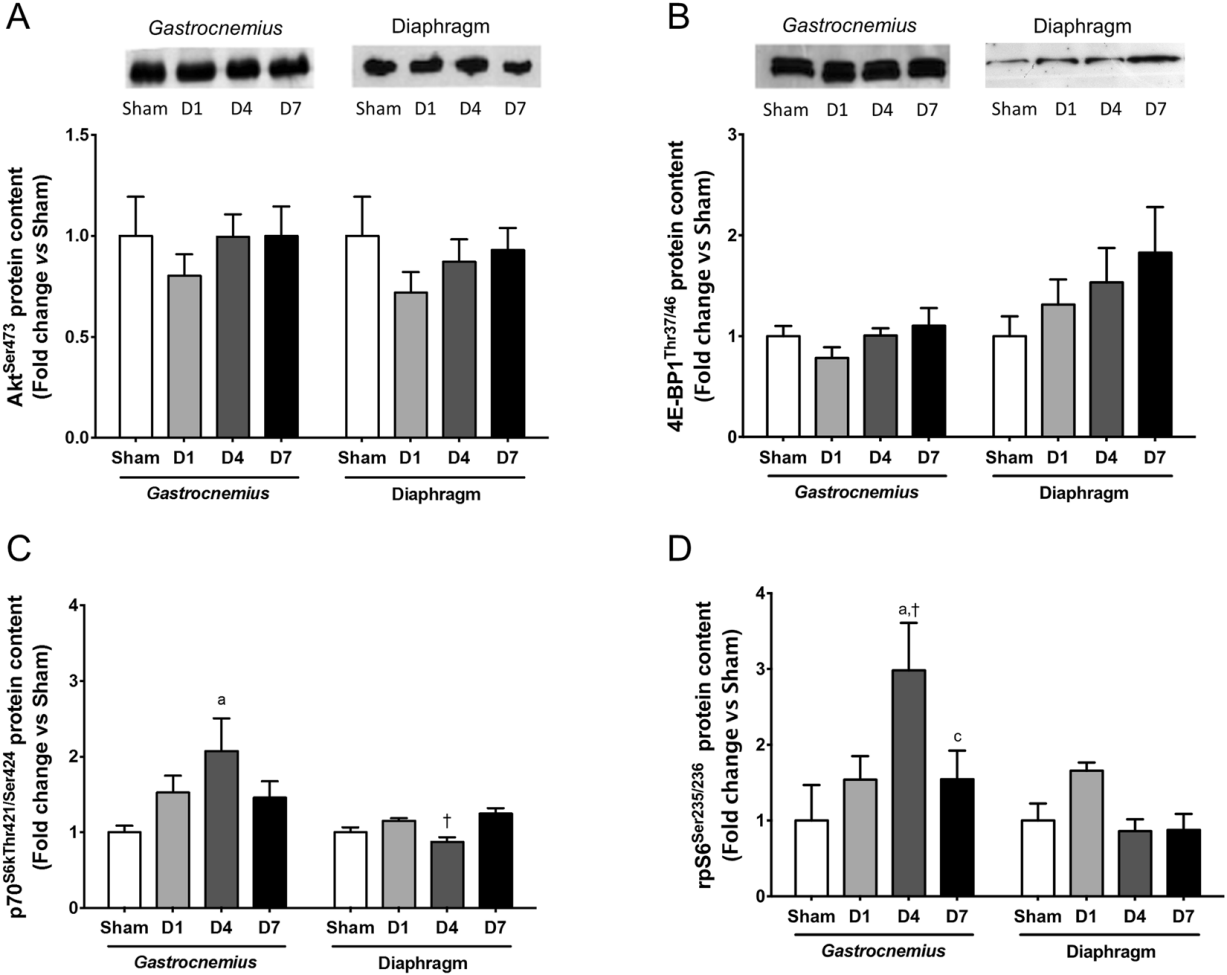
Regulation of IGF-1-Akt-mTOR pathway in *gastrocnemius* and diaphragm muscles. Immunoblot analysis of Akt phosphorylation on Ser473 (upper panel) and quantification of phosphorylated protein level (lower panel) (**A**). Immunoblot analysis of 4E-BP1 phosphorylation on Thr37 and Thr46 (upper panel) and quantification of phosphorylated protein level (lower panel) (**B**). Luminex analysis of p70^S6K^ phosphorylation on Thr421 and Ser424 (**C**). Luminex analysis of rpS6-BP1 phosphorylation on Ser235 and Ser236 (**D**). D1: one day after CLP; D4: 4 days after CLP; D7: 7 days after CLP. All data are normalized to sham animals. Data are expressed as means ± SEM (n = 8/group). a: significantly different from Sham mice; c: significantly different from D4; †: significantly different from *gastrocnemius* muscle at the same time point.

Sepsis can trigger muscle wasting by regulating the balance between protein synthesis and degradation. We first explored the regulation of the IGF-1-Akt-mTOR pathway, a crucial regulator of skeletal muscle protein synthesis whose activation prevents muscle atrophy *in vivo* ^33,34^. Although the phosphorylation level of Akt and 4E-BP1 remained unchanged in the *gastrocnemius* muscle after sepsis (Fig. 3), both p70^S6k^ and rpS6 phosphorylation were significantly increased 4 days after CLP in *gastrocnemius* muscle. No change was observed in diaphragm muscle. Total protein level of Akt and 4E-BP1 also remained unchanged (Supplementary Fig. 3).

### The ubiquitin-proteasome system is differentially regulated in *gastrocnemius* and diaphragm muscles during sepsis

**Figure 4:**
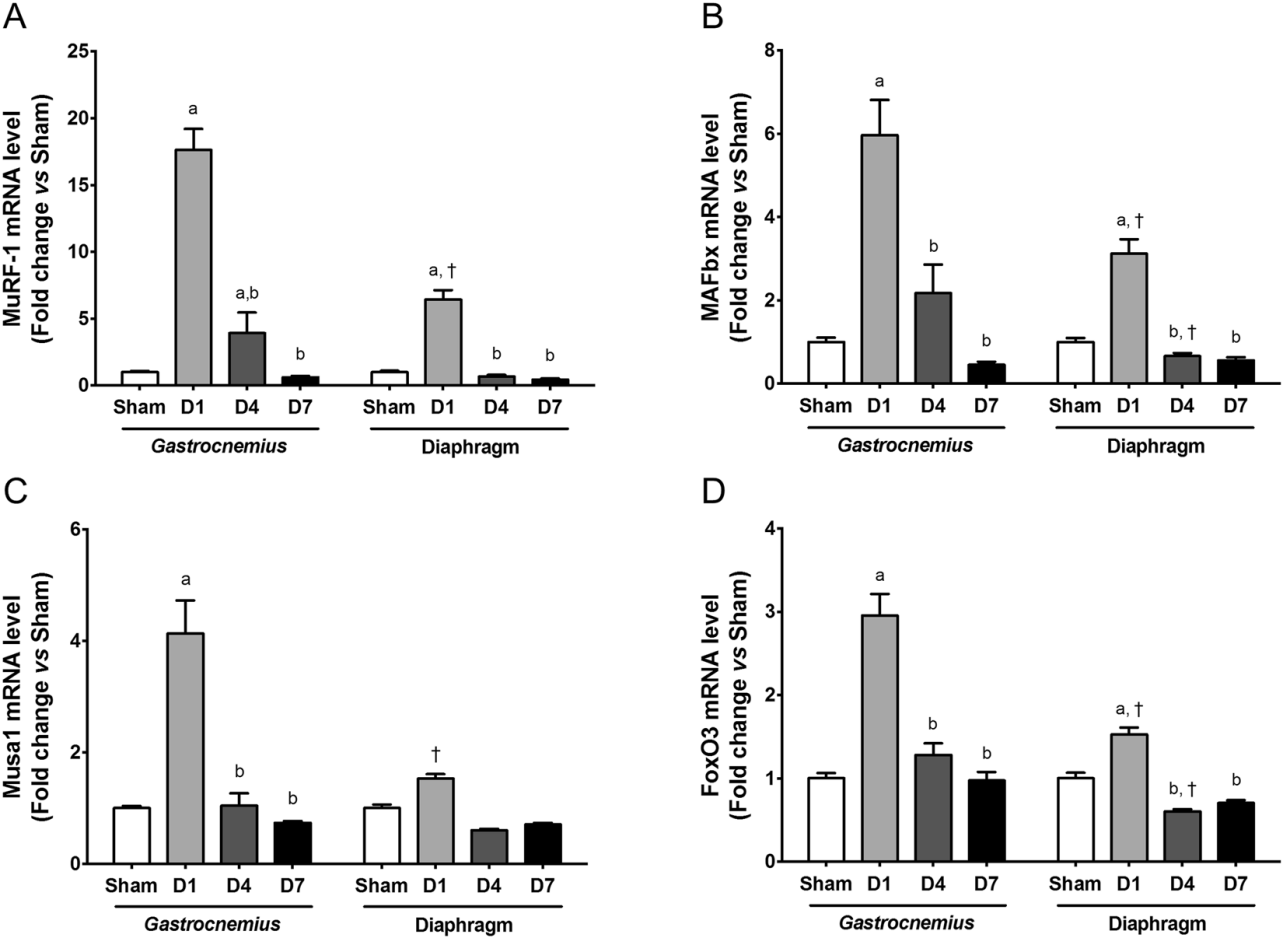
mRNA levels of atrogens and FoxO3a in *gastrocnemius* and diaphragm muscles. Transcript levels of MuRF-1 (**A**), MAFbx (**B**), Musa1 (**C**) and FoxO 3a (**D**). D1: one day after CLP; D4: 4 days after CLP; D7: 7 days after CLP. All transcript levels are expressed relative to sham values. Data are expressed as means ± SEM (n = 8/group). a: significantly different from Sham; b: significantly different from D1; †: significantly different from muscle at the same time point.

The mRNA levels of the E3-ubiquitin ligases, muscle RING finger-1 (MuRF-1), muscle atrophy F-box (MAFbx) and muscle ubiquitin ligase of SCF complex in atrophy-1 (Musa 1) involved in ubiquitin-proteasome-dependent proteolysis^16,33,35^ were transitory increased one day after CLP both in *gastrocnemius* muscle and diaphragm muscle (Fig. 4A–C). However, the extent of the transcriptional response was significantly higher in *gastrocnemius* muscle than in diaphragm muscle. Whereas expression of MuRF-1 increased by about 18-fold one day after CLP in *gastrocnemius* muscle, MuRF-1 expression increased by 6-fold in diaphragm muscle (p < 0.005). Similarly, expression of MAFbx, which was increased by about 6-fold in *gastrocnemius* muscle, was only increased by about 3-fold in diaphragm muscle (*p* < 0.05) one day after CLP. The expression profile of MuRF-1, MAFbx and Musa 1 in *gastrocnemius* and diaphragm muscles was also closely related to the expression profile of FoxO3, a transcription factor involved in the transcriptional regulation of these E3-ubiquitin ligases^36^ (Fig. 4D). Collectively, all these findings suggest that sepsis activates the ubiquitin-proteasome pathway in locomotor and respiratory muscles, but that the extent of activation is greater in locomotor muscles.

**Figure 5:**
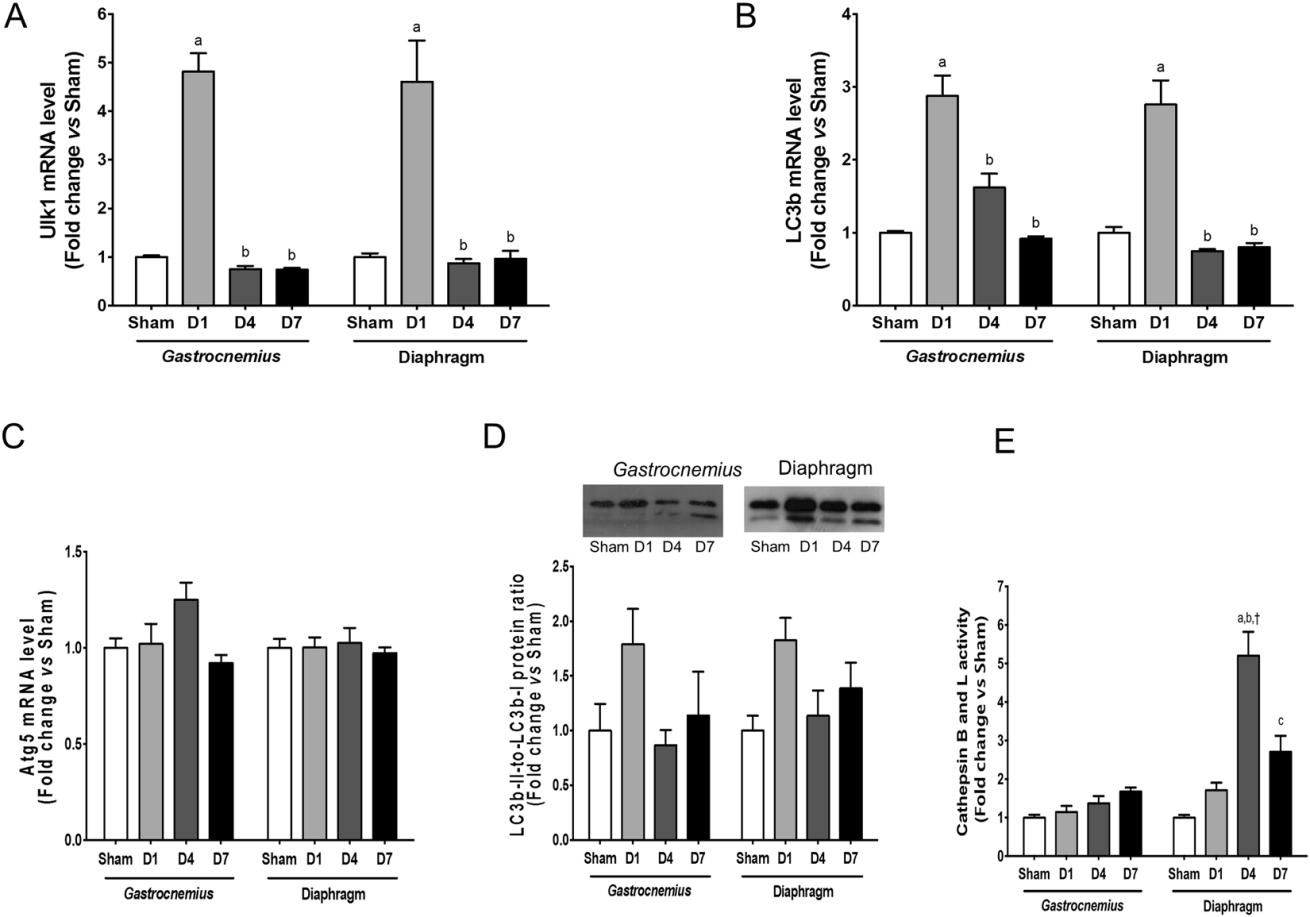
Regulation of autophagy-lysosome pathway in *gastrocnemius* and diaphragm muscles. Transcript level of Ulk1 (**A**), LC3b (**B**) and Atg5 (**C**). Immunoblot analysis of LC3b1 and LC3b2 (upper panel) and quantification of LC3b1-to-LC3b2 ratio (lower panel) (**D**). Enzyme activity of cathepsin B and cathepsin L (**E**). D1: one day after CLP; D4: 4 days after CLP; D7: 7 days after CLP. All data are expressed relative to Sham values. Data are expressed as means ± SEM (n = 8–11/group). a: significantly different from Sham; b: significantly different from D1; c: significantly different from D4; †: significantly different from *gastrocnemius* muscle at the same time point.

We also monitored the expression of autophagy-related genes. Whereas Atg5 mRNA level remained unchanged in both muscles, mRNA levels of Ulk1 and LC3b were all significantly increased one day after CLP in both muscles (Fig. 5A–C). Similarly, LC3b-II to-LC3b-I ratio was non-significantly increased in *gastrocnemius* and diaphragm muscles one day after CLP (Fig. 5D). Finally, the enzyme activity of the lysosomal enzymes, cathepsin B and L, was only increased in diaphragm muscle 4 days after CLP (Fig. 5E). Therefore, sepsis regulates similarly the expression of autophagy-related genes in *gastrocnemius* and diaphragm muscles, but differentially regulates lysosomal activity.

### Regulation of Smad2/3 and Smad1/5/8 signaling pathways in *gastrocnemius* and diaphragm muscles during sepsis

**Figure 6:**
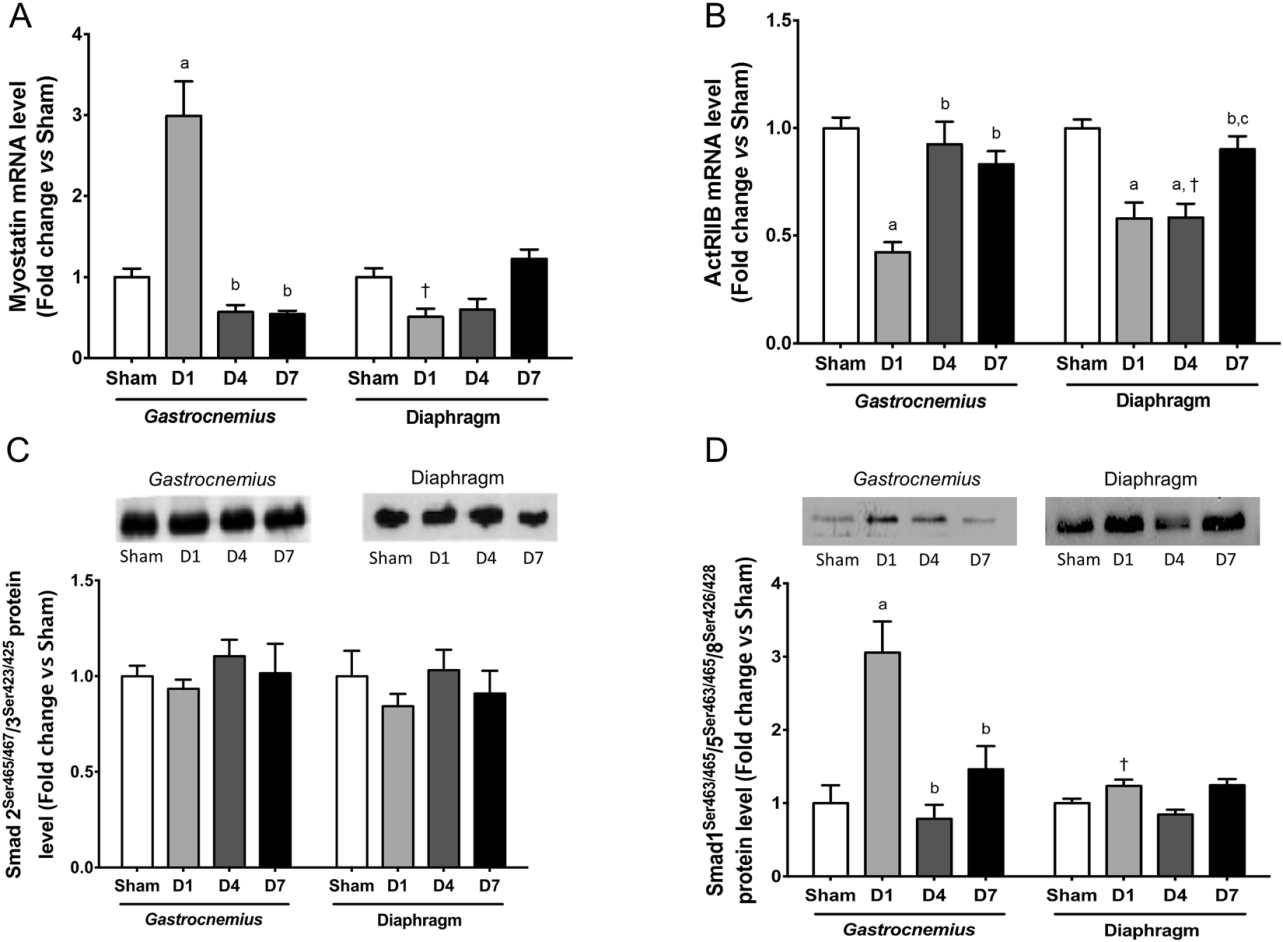
Expression of myostatin and phosphorylation of Smad1/5/8 in *gastrocnemius* and diaphragm muscles. Transcrip level of myostatin (**A**) and activin type IIb receptor (**B**). Immunoblot analysis of Smad2/3 phosphorylation on Ser423 and Ser425 (upper panel) and quantification of Smad2/3 phosphorylation level (lower panel) (**C**). Immunoblot analysis of Smad1/5/8 phosphorylation on Ser463, Ser465, Ser 426 and Ser428 (upper panel) and quantification of Smad1/5/8 phosphorylation level (lower panel) (**D**). D1: one day after CLP; D4: 4 days after CLP; D7: 7 days after CLP. All data are expressed relative to sham values and presented as means ± SEM (n = 7–8/group). a: significantly different from Sham; b: significantly different from D1; c: significantly different from D4; †: significantly different from *gastrocnemius* muscle at the same time point.

Myostatin mRNA level was only markedly increased by about 3-fold one day after CLP specifically in *gastrocnemius* muscle (Fig. 6A). Myostatin acts in an autocrine/paracrine manner by binding to the activin type IIB receptor (ActRIIB). The transcript level of ActRIIB was decreased one day after CLP in *gastrocnemius* muscle, whereas this decrease was maintained 4 days after CLP in diaphragm muscle (Fig. 6B). ActRIIB then recruits and activates type-I activin receptors, which in turn causes the phosphorylation of Smad2 and Smad3. Here, the phosphorylation level of Smad2/3 remained unchanged in *gastrocnemius* and diaphragm muscles (Fig. 6C).

Bone morphogenetic proteins (BMPs)-Smad1/5/8 signaling pathway is an important positive regulator of skeletal muscle mass^16,17^. BMPs bind to dedicated BMP receptors (Alk3, Alk6) that in turn phosphorylate Smad1/5/8 proteins. Activation of this pathway is consistently observed during muscle atrophy to prevent excessive muscle mass loss^16,18^. In agreement with these data, Smad1/5/8 phosphorylation was only increased in *gastrocnemius* muscle one day after CLP (Fig. 6D).

### Expression of inflammatory cytokines is increased in diaphragm muscle but not in *gastrocnemius* muscle during sepsis

**Figure 7:**
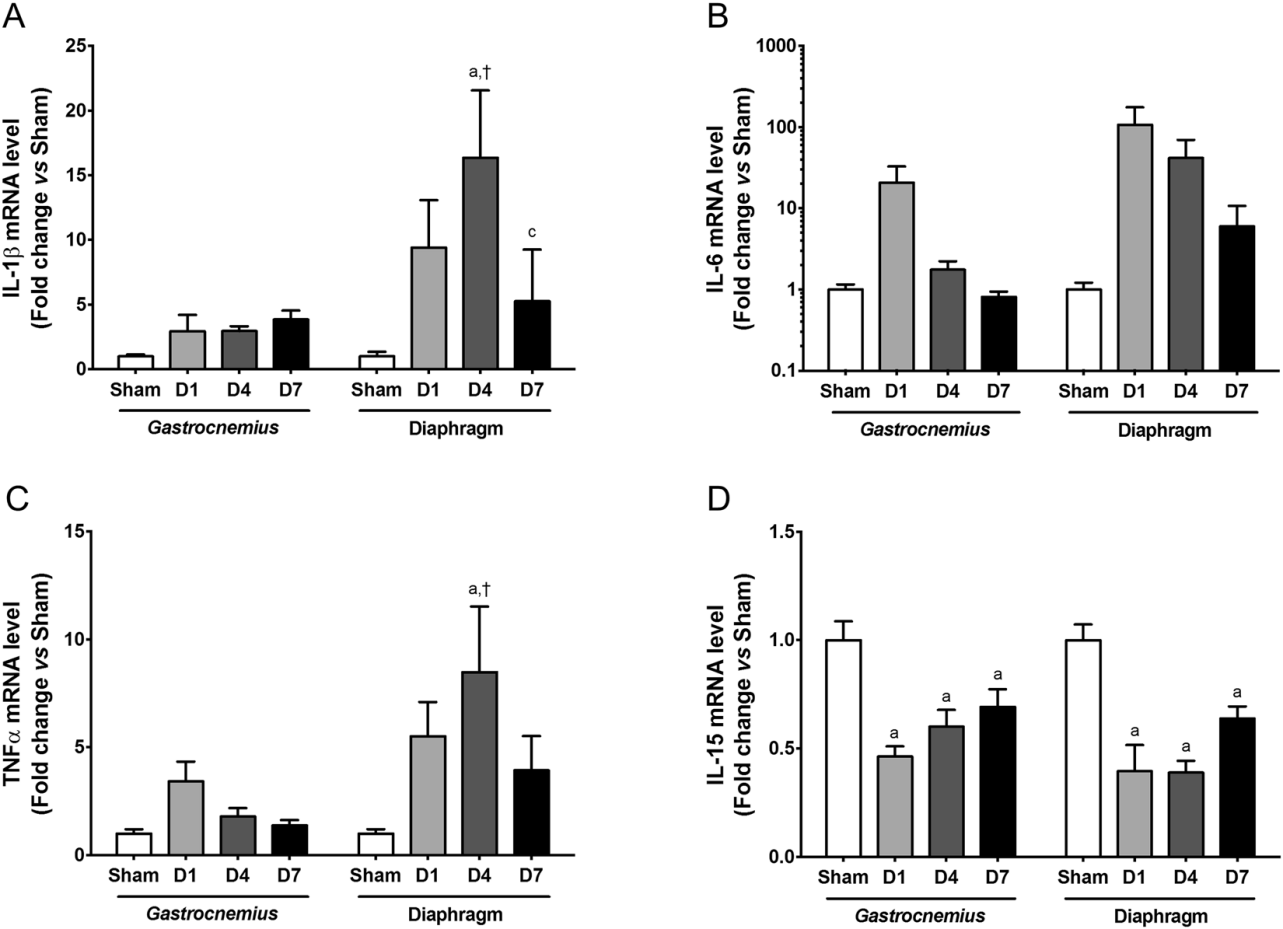
Expression of pro- and anti-inflammatory cytokines in *gastrocnemius* and diaphragm muscles. Effect of sepsis on IL-1β (**A**), IL-6 (**B**), TNF-α (**C**) and IL-15 (**D**) transcript levels in *gastrocnemius* and diaphragm muscles. D1: one day after CLP; D4: 4 days after CLP; D7: 7 days after CLP. Data are expressed relative to sham values and presented as means ± SEM (n = 7–8/group). a: significantly different from Sham; c: significantly different from D4; †: significantly different from *gastrocnemius* muscle at the same time point.

A local inflammatory response could promote atrophy of skeletal muscle during sepsis. The mRNA levels of pro-inflammatory (IL-1β, IL-6 and TNF-α) and anti-inflammatory (IL-15) genes were thus determined in the *gastrocnemius* and diaphragm muscles of mice. In agreement with a previous report^37^, there was a higher transcript level of pro-inflammatory cytokines in diaphragm muscle compared to *gastrocnemius* muscle in the sham condition (Supplementary Fig. 4). Although some differences in the kinetic response of the pro-inflammatory genes were observed, the extent of the response was significantly greater in the diaphragm muscle than in the *gastrocnemius* muscle (Fig. 7A–C). Finally, the transcript level of the anti-inflammatory cytokine, IL-15, was significantly decreased from day 1 after CLP to day 7 both in *gastrocnemius* and diaphragm muscles (Fig. 7D).

## Discussion

ICU-acquired muscle weakness is associated with prolonged mechanical ventilation, longer length of stay and increased mortality, but also long term functional disabilities. Sepsis is the most common risk factor of ICU-acquired muscle weakness. In the present study, we used CLP, a relevant model of sepsis-induced muscle wasting^38^ to explore the molecular mechanisms involved in the control of muscle mass in locomotor and respiratory muscles.

Atrophy of locomotor muscle was clearly evident after CLP. Accordingly, previous studies indicate that atrophy of locomotor muscles is an early event consistently observed during sepsis that persists at least 2 weeks after the induction of sepsis^26,31,39,40^. Multiple factors may contribute to trigger muscle mass loss, including alteration in nerve impulse, malnutrition and disuse. ICU-acquired weakness is associated with a reduction in the amplitude of motor and sensory nerve action potentials with normal or mildly reduced nerve conduction velocity^5^. Biopsies from ICU patients revealed a primary distal axonal degeneration of motor and sensory fibers^41^. Alterations in skeletal muscle innervation may contribute to the atrophy of *gastrocnemius* muscle after sepsis, this has not been explored here. Sepsis has been shown to induce hypophagia early during the infectious episode^31^. In the present study, sham animals were pair-fed to septic mouse, thus excluding the possibility that skeletal muscle atrophy was due to starvation, suggesting that sepsis-induced muscle mass loss is independent of hypophagia. Another important factor to consider is disuse. Septic mice were prostrated when compared to sham animals, strongly suggesting a reduced daily locomotor activity in these animals and could have contributed to increase muscle atrophy through the mechanical silencing mechanism^42^. However, muscle atrophy in critically ill patients has been shown to exceed that observed in bed-ridden patients^43^. Therefore, even if muscle disuse is an important factor that contributes to skeletal muscle atrophy in response to sepsis, other factors directly associated with the septic episode probably worsen atrophy of locomotor muscles.

One main feature of the present study was the different response of the *gastrocnemius* and diaphragm muscles to sepsis, the mass of diaphragm muscle being completely preserved 7 days after CLP. This contrasts with previous data showing a preferential weakness of the diaphragm muscle compared to locomotor muscles in an animal model of sepsis induced by pulmonary infection^44,45^. However, the specificity of the infectious model (chronic pulmonary infection) may explain the atrophy of the diaphragm muscle in these studies. Similarly, sepsis has been shown to be a worsen condition that further increases diaphragm atrophy in mechanically ventilated ICU patients^7^. In the present study, maintenance of a spontaneous contractile activity of the diaphragm muscle could have protected diaphragm muscle against sepsis-induced atrophy. Diaphragm muscle activation is stereotypical in that it undergoes phasic activation and stretching during the breathing cycle throughout life. The daily duty cycle (ratio of active to inactive times) for hind limb muscles ranges from 2% for the *extensor digitorum longus* muscle to 14% for the *soleus* muscle, whereas that of the diaphragm muscle is about 45%^46^. The unique activation pattern of diaphragm muscle could thus explain the preservation of diaphragm muscle mass in the present study. Furthermore, under septic conditions without mechanical support of ventilation, respiratory rate also increases as a consequence of both the activation of central nervous system and an increase in peripheral metabolic demand^47^. This could also contribute to maintain/increase the mechanical load of diaphragm muscle during experimental sepsis. Taken together, these data suggest that chronic stimulation of skeletal muscle could be a strategy to limit or prevent the loss of skeletal muscle after CLP. In patients with sepsis, these data also support the idea that early rehabilitation would be an efficient intervention to counteract the loss of muscle mass.

The observation that muscle atrophy only occurred in *gastrocnemius* muscle also suggests the existence of a different regulation pattern of signaling pathways involved in the control of muscle mass between *gastrocnemius* and diaphragm muscles. Mechanistically, *gastrocnemius* muscle atrophy did not involve a significant down-regulation of IGF-1-Akt-mTOR pathway. By contrast, the increase in rpS6 and p70S6k phosphorylation 4 days after CLP rather suggests that the anabolic pathway is activated to limit the extent of *gastrocnemius* muscle atrophy^16^. This response was not observed in the diaphragm muscle. Our data also suggest the ubiquitin-proteasome dependent proteolysis is strongly activated in the *gastrocnemius* muscle of septic mice, as evidenced by the expression of the E3-ubiquitin ligases MuRF1, MAFbx and Musa1. Accordingly, transcript level of FoxO, a transcription factor regulating the expression of these E3-ubiquitin ligases^36^, showed the same expression profile. Although this molecular signature was also observed in the diaphragm muscle, the extent of the transcriptional response was significantly lowered in the diaphragm muscle compared to the *gastrocnemius* muscle. This strongly suggests that the resistance of the diaphragm muscle to sepsis-induced atrophy could be mediated by a lower expression of these E3-ubiquitin ligases and therefore a lower activation of ubiquitin-proteasome system. The ubiquitin-proteasome system has been shown to increase protein breakdown in locomotor muscle early during sepsis both in human patients^23,48^ and in animal models of sepsis^26,31,49,50^. MAFbx promotes the degradation of MyoD^51^, a muscle transcription factor involved in the expression of muscle-specific genes, and eIF3-f (eukaryotic initiation factor-3f), an activator of translation initiation^52^, whereas MURF1 catalyses the ubiquitination and degradation of myofibrillar proteins, including troponin^53^ and myosin heavy chains^54^, myosin light chains^55^ and actin^56^. Therefore, an increase in MuRF-1 and MAFbx expression could contribute to decrease the expression of muscle-specific genes and protein translation.

The autophagy-lysosome system is involved in skeletal muscle atrophy induced by denervation or fasting^14,15^. But autophagy also plays a critical role for myofiber maintenance, and its activation is necessary to avoid the accumulation of dysfunctional organelles and toxic proteins that would lead to muscle atrophy and weakness^57^. Therefore, autophagy can be either viewed as beneficial or detrimental for the maintenance of muscle mass. Both *gastrocnemius* and diaphragm muscles displayed a transitory increase (day 1) in the transcript level of autophagy-related genes (Ulk1, LC3b), together with an increase in LC3b-II-to-LC3b-I ratio, suggesting that autophagosome formation would be increased in response sepsis. Accordingly, earlier clinical or experimental studies have reported an enhanced expression of autophagy-related genes and autophagy proteins during sepsis^23,45^. However, this interpretation has to be relativized, since we did not measure autophagy flux to evidence the formation of autophagosomes and their subsequent fusion with lysosomes^58^. Furthermore, an increase activity of cathepsin B and cathepsin L was only observed in diaphragm muscle, suggesting that downstream of autophagosome formation, the degradation of autophagosome content by lysosomal proteases could be also increased in diaphragm muscle. Therefore, the lysosomal response of the diaphragm muscle could be thus beneficial to avoid the accumulation of dysfunctional organelles and toxic proteins that would lead to muscle atrophy and weakness.

Our data suggest that myostatin is a regulator of locomotor muscle mass during sepsis. Once again diaphragm muscle displayed a completely different response. Whereas, myostatin transcript level was transitory increased by about 3-fold in *gastrocnemius* muscle one day after CLP, myostatin mRNA level remained unchanged in diaphragm muscle, suggesting that this differential response may contribute to preserve the mass of diaphragm muscle during sepsis. Surprisingly, Smad2/3 phosphorylation was unchanged in *gastrocnemius* muscle. However, extensive activation of myostatin signaling has been shown to trigger the expression of Smad7^59^, which in turn represses Smad2/3 phosphorylation^60^. Together with the down-regulation of ActR2B transcript level, such a mechanism could thus limit the extent of atrophy in *gastrocnemius* muscles. Activation of the BMP signaling has been shown to prevent excessive muscle mass loss in response to denervation and fasting^16,17^. Accordingly, this pathway was only activated in *gastrocnemius* muscle, which is consistent with the observation that muscle mass loss occurred in *gastrocnemius* muscle but not in diaphragm muscle. Activation of the BMP signaling pathway has been shown to repress Musa1 expression^16^ and the transcriptional activity of myogenin on MuRF1 and MAFbx promoters^17^. The reported increase in MuRF1, MAFbx, and Musa1 expression in the *gastrocnemius* muscle indicates that other regulatory influences counteract BMP signaling. Overall, these data indicate that the coexistence of anabolic and catabolic pathways that are contemporally activated in response to sepsis.

Activation of the innate immune system by sepsis produces pro-inflammatory cytokines (TNFα, IL-1β and IL-6) that may trigger muscle wasting^61,62^. Skeletal muscle also produces cytokines that locally may further amplify the cytokinic response. Interestingly, we observed a concomitant increase in macrophage content in the gastrocnemius muscle of CLP mice. This is consistent with the presence of macrophages in 40% of muscle biopsies from patients who developed critical illness polyneuropathy and myopathy^63^. In the present study, TNFα, IL-1β and IL-6 transcript levels were all increased in *gastrocnemius* and diaphragm muscles during sepsis, but this response was significantly greater and more sustained over time in the diaphragm muscle. An increase in the expression of TNFα, IL-1β and IL-6 has been shown *in vitro* to inhibit protein synthesis and to increase proteolysis^62^. Paradoxically, muscle mass loss prevailed in *gastrocnemius* muscle. Therefore, one may consider that the diaphragm muscle is relatively predisposed to trigger a pro-inflammatory response^37^ and that an acute and vigorous inducible inflammatory response timely resolved after sepsis is not detrimental for diaphragm muscle, but rather beneficial by preventing excessive muscle mass loss.

**Figure 8:**
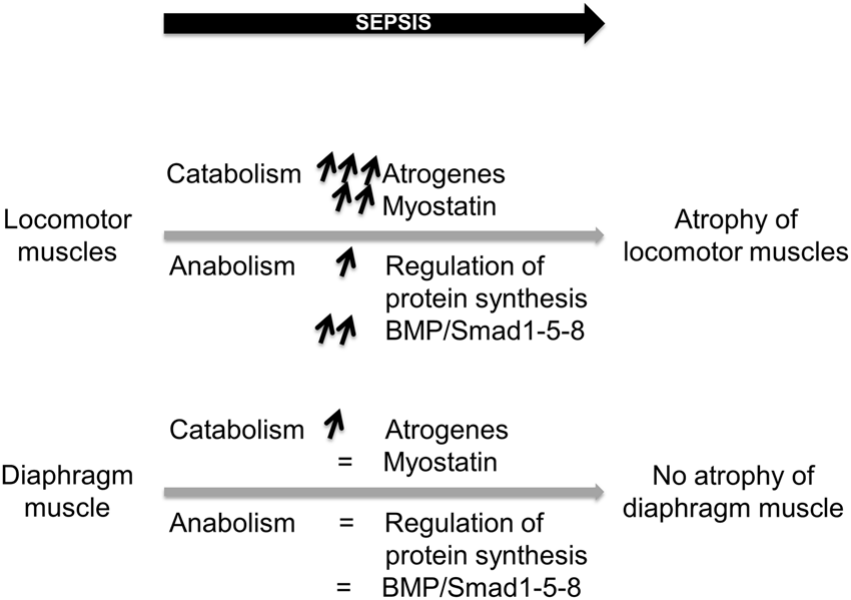
Graphical abstract of the study. Locomotor muscles are more prone to sepsis-induced muscle mass loss compared to the diaphragm respiratory muscle. Mechanistically, this could be explained by a larger increase in the expression of atrogenes (MuRF-1, MAFbx, Musa1) and by a strong increase in myostatin expression. Activation of anabolic pathways (Akt-mTOR and BMP/Smad1-5-8) in locomotor muscles is interpreted as a compensatory response triggered to limit the extent of sepsis-induced muscle atrophy. Physiologically, maintaining and/or increasing the mechanical load of diaphragm muscle during experimental sepsis could contribute to preserve diaphragm muscle from atrophy.

In conclusion, we report here that induction of severe sepsis by CLP in mice elicits an atrophy of locomotor muscles, which is not found in diaphragm respiratory muscle (Fig. 8). Preventing muscle atrophy during sepsis through early rehabilitation would be an important outcome to improve post-sepsis recovery and patients’ life quality.

## Materials and Methods

### Animal model

All experiments were conducted in accordance with the European Community guidelines for the care and use of laboratory animals for scientific purposes, and after the authorization of the Ministère de l’Education Nationale, de l’Enseignement supérieur et de la Recherche and the Comité d’Ethique en Expérimentation Animale de la Loire (CEEA 98, Université Jean Monnet, N° CU13N6). Sepsis was induced by cecal ligation and puncture (CLP) in 13-week-old C57BL6/J male mice (Janvier Labs S.A.S. France) (n = 50). Under isoflurane anesthesia (2.5% in air) and analgesia (subcutaneous injection of 0.05 mg.kg^−1^ buprenorphine), the cecum was exposed, ligatured and punctured one time with a 18.5 G needle. The cecum was then carefully replaced into the abdomen, and the abdomen wall and the skin were sutured^64^. Sham C57BL6/J male mice (n = 16) were subjected to the same surgical procedure except that the cecum was neither tied nor punctured. Shortly after surgery and during 3 days, animals received physiological saline (1 ml per 10 g of body weight) subcutaneously. Mice from the sham group were pair-fed to septic animals. Mice body weight was recorded every morning.

### Muscle force

After acclimatization with the device, maximal peak force of both forelimb and hindlimb was assessed the day before the surgery and 2, 4 and 7 days after CLP by using a grip test (Bio-GS3, Bioseb, France). Peak force was recorded on 3 consecutive trials and the best of 3 consecutive trials was used for analysis.

### Tissue removal

Tissues from septic animals were removed one day (n = 11), 4 days (n = 9) and 7 days (n = 11) after CLP. Tissues from sham animals were removed one day (n = 8) and 7 days after surgery (n = 8). No significant difference was observed for any of the variables that were studied in sham animals. All sham animals have been therefore gathered together in one unique sham group. Mice were anesthetized (*i.p*. injection of 90 mg.kg^−1^ ketamine and 10 mg.kg^−1^ xylazine). *Extensor digitorum longus*, *gastrocnemius*, *soleus, quadriceps, tibialis anterior* and diaphragm muscles were rapidly excised, weighed and snap frozen. The *gastrocnemius* muscle was mounted in embedding medium and frozen in thawing isopentane for histological analyses. Subcutaneous fat and spleen were also removed and weighed. Mice were then killed by exsanguination.

### Immunohistomorphometry

*Gastrocnemius* muscles were cut (12 µm) in a refrigerated (−20 °C) cryostat (Microm HM 560). For the determination of muscle fiber cross-sectional area^18^, transverse sections were fixed in phosphate buffer saline containing 4% paraformaldehyde and incubated with anti-laminin (1:200; Sigma-Aldrich, Saint-Quentin Fallavier, France). Nuclei were counterstained with 4′,6-diamidino-2-phenylindole. Fluorescent muscle fibers were visualized using a confocal scanning laser inverted microscope (Leica TCS-SP2, Heidelberg, Germany). The cross-sectional area of 1062 ± 309 fibers per muscle was quantified using ImageJ software (http://rsb.info.nih.gov/ij/). For the quantification of macrophages in skeletal muscle, cryosections were labeled with antibodies against F4/80 (#ab6640, Abcam) and laminin (#L9393, Sigma Aldrich) overnight at 4 °C and labeling using the second antibody was performed for 2 hours at 37 °C. Secondary antibodies were coupled to Cy3 and FITC (Jackson Immunoresearch Inc), respectively. Fluorescent immunolabelings were recorded with a Zeiss Axio Observer A1 microscope connected to a CoolSNAP HQ^2^ camera at 20X magnification. For each experiment, at least 5 randomly chosen fields were counted in a blinded manner (range: 5–14 fields, mean: 9 ± 3 fields). The number of labeled macrophages (*i.e*., F4/80 positive cells) was calculated using Image J software (http://rsb.info.nih.gov/ij/) and normalized to the analyzed muscle area.

### RNA isolation, cDNA synthesis and real time quantitative polymerase chain reaction

Total RNA was collected from *gastrocnemius* muscle by using a phenol/chloroform extraction (Qiazol, Qiagen) followed by column purification (NucleoSpinRNA, Macherey Nagel). RNA (200 ng) was reverse transcribed using the iScript cDNA synthesis kit (Bio-Rad). The selected forward and reverse primer sequences are listed in Supplementary Table 1. Real-time PCR was performed in a 20 µl final volume using the SsoFast EvaGreen Super mix (Bio-Rad). Fluorescence intensity was recorded using a CFX96 Real-Time PCR Detection System (Bio-Rad). Data were analyzed using the ∆∆CT method of analysis. Reference genes (hypoxanthine guanine phosphoribosyl transferase, ribosomal protein large P0 and α-tubulin) were used to normalize the expression levels of genes of interest^65^.

### Protein extraction

Powdered *gastrocnemius* muscles were homogenized (1:20 dilution wt:vol) in an ice-cold 50 mM Tris HCl buffer (pH 7.4) containing 100 mM NaCl, 2 mM EDTA, 2 mM EGTA, 50 mM β-glycerophosphate, 50 mM sodium fluoride, 1 mM sodium orthovanadate, 120 nM okadaic acid, and 1% Triton X-100. Muscle extracts were then centrifuged at 12,000 g for 10 min at 4 °C. Protein concentration of the supernatant was determined at 750 nm (Bio-Rad Protein Assay kit).

### Immunoblotting

Fifty µg of proteins were resolved on 12.5% SDS-polyacrylamide gels, and then blotted onto 0.45 µm nitrocellulose membranes (GE Healthcare Life Sciences), and incubated overnight with the appropriate antibody. Antibodies against phosphorylated Smad 2Ser465/467-3 Ser423/425 (1:1,000), Smad1Ser463/465-5Ser463/465-8Ser426/428 (1:500 vol/vol), Akt (1:2,000), phosphorylated AktSer473 (1:1,000), 4EBP1 (1:5,000), phosphorylated 4EBP1-PThr37/46 (1:1,500), p70S6k (1:1,000) and α-tubulin (1/2,000) were from Cell Signaling Technology. Antibody against LC3b (1:500) was from Sigma-Aldrich. Incubation with horseradish peroxidase-conjugated secondary antibody (Dako) allowed the chemiluminescent detection of immunocomplexes (GE Healthcare Life Sciences). α-tubulin immunoblots were used to check for equal protein loading. Labeled membranes were quantified using ImageJ software (http://rsb.info.nih.gov/ij/).

### Luminex analyses

Fluorescent capturing beads coupled to antibodies directed against phosphorylated p70^S6kThr421/Ser424^ and phosphorylated ribosomal protein S6 rpS6^Ser235/236^ (Bio-Rad) were incubated overnight with 50 µl of protein fractions (1:10 dilution vol:vol) in 96-well plates. Samples were then washed, incubated with biotinylated antibodies for 30 min, followed by the incubation with a streptavidin-phycoerythrin solution for 10 min. The analysis consisted of a double-laser fluorescence detection, which allowed simultaneous identification of the target protein through the red fluorescence emission signal of the bead and quantification of the target protein through the fluorescence intensity of phycoerythrin. Fluorescence intensities were recorded on a Bio-Plex™ 200 System instrument (Bio-Rad). Data were analyzed using Bio-Plex Manager software.

### Enzyme activities

Fluorescent recording of chymotrypsin-like activity from the 20 S subunit of the proteasome (λ_exc = _380 nm and λ_em_ = 460 nm), cathepsin B + L activity (λ_ex_ = 380 nm and λ_em_ = 460 nm) and citrate synthase activity (λ_ex _ =340 nm and λ_em_ = 450 nm) was determined using SFM25 fluorimeter (Kontron instruments) as previously described^66^.

### Statistical Analysis

Animals were randomly assigned to a sham group or a sepsis group. All values are expressed as mean ± SEM. A two-way ANOVA was used to analyze the kinetic of body weight variation after surgery (Statsoft®). For the analysis of the changes in muscle force as a function of time, tissue weight and immunohistomorphometry, a one-way ANOVA was used to determine differences between groups (CLP and Sham) as a function of time (1, 4 and 7 days after CLP). A two-way ANOVA was used for the comparison of other variables between *gastrocnemius* and diaphragm muscles using Newman-Keuls *post hoc* test. The α-level of significance was set at 0.05.

## Electronic supplementary material

supplemental data
