# Supplementary material for: Regulation of Akt-mTOR, ubiquitin-proteasome and autophagy-lysosome pathways in locomotor and respiratory muscles during experimental sepsis in mice
Source: Sci Rep. 2017 Sep 7;7:10866. doi: 10.1038/s41598-017-11440-5 (PMC5589872; doi:10.1038/s41598-017-11440-5)
Supplement: Supplementary file 1 — supplemental data [file 41598_2017_11440_MOESM1_ESM.doc]

Regulation of Akt-mTOR, ubiquitin-proteasome and autophagy-lysosome pathways in locomotor and respiratory muscles during experimental sepsis in mice.

Jérome Morel^1,2^, Jean-Charles Palao^1,2^, Josiane Castells^1^, Marine Desgeorges^1^, Thierry Busso^1^, Serge Molliex^2^, Vanessa Jahnke^1^, Peggy Del Carmine^3^, Julien Gondin^3^, David Arnould^1^, Anne Cécile Durieux^1^, Damien Freyssenet^1^

^1^Univ Lyon - University Jean Monnet Saint Etienne ; Inter-university Laboratory of Human Movement, EA7424, F-42023, Saint Etienne, France.

^2^Département d’anesthésie et réanimation, Centre Hospitalier Universitaire de Saint Etienne, Saint Etienne, France.

^3^Institut NeuroMyoGène, Université Claude Bernard Lyon 1, INSERM U1217, CNRS UMR 5310 Villeurbanne, France.

Address for correspondence: Jérome Morel

Laboratoire Interuniversitaire de Biologie de la Motricité, Faculté de Médecine,

10 rue de la Marandière

42270 Saint Priest en Jarez, France

[jerome.morel@chu-st-etienne.fr](mailto:jerome.morel@chu-st-etienne.fr)

**Supplementary Figure 1.** Survival rate of Sham mice after surgery (dotted line; n= 20 before surgery) and septic mice (full line; n= 40 before CLP). The survival rate of septic mice was significantly different (p < 0.05) from Sham mice.


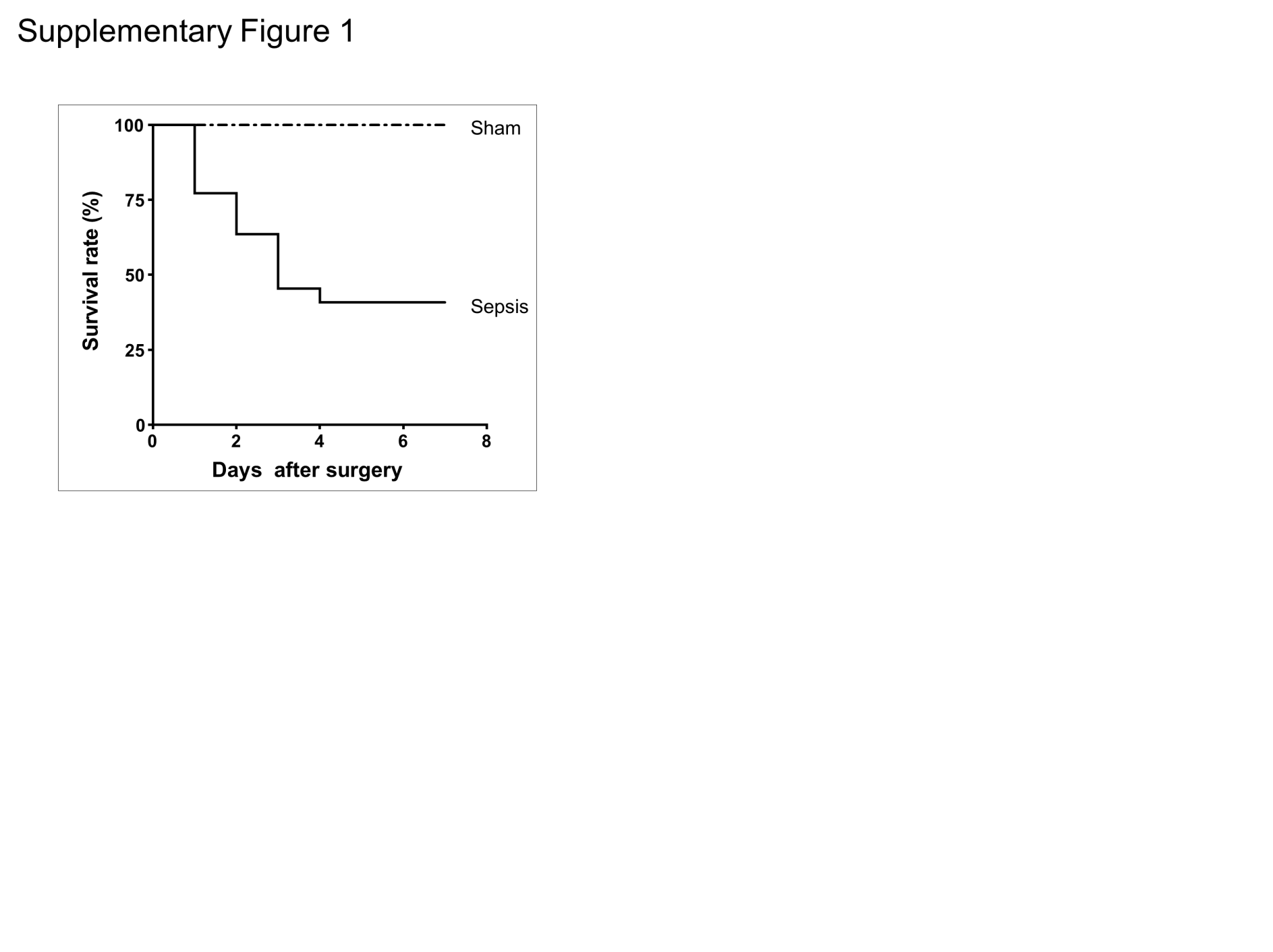


**Supplementary Figure 2.** Skeletal muscles weight of hindlimb locomotor muscles.

Weight of *extensor digitorum longus* (**A**), quadriceps (**B**), *soleus* (**C**) *tibialis anterior* and (**D**) muscles. D1: one day after CLP; D4: 4 days after CLP; D7: 7 days after CLP. Data are expressed as means ± SEM (n= 9/11 group). a: significantly different from Sham; b: significantly different from D1; c: significantly different from D4.

**
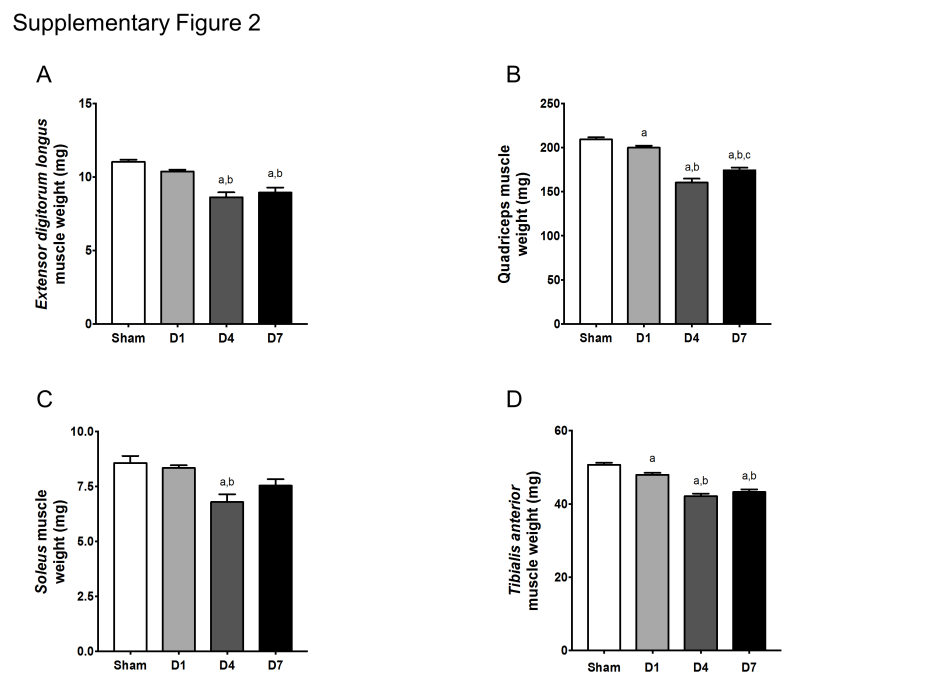
**

**Supplementary Figure 3**

Total forms of Akt, 4EB-P1 and p70^S6K^ remained unchanged in *gastrocnemius* and diaphragm muscles in response to sepsis. Immunoblot analysis of Akt (upper panel) and quantification of total protein level (lower panel) (**A**). Immunoblot analysis of 4E-BP1 (upper panel) and quantification of total protein level (lower panel) (**B**). Immunoblot analysis of p70^S6K^ and quantification of total protein level (lower panel) (**C**). D1: one day after CLP; D4: 4 days after CLP; D7: 7 days after CLP. Data are expressed as means ± SEM (n= 8/group).


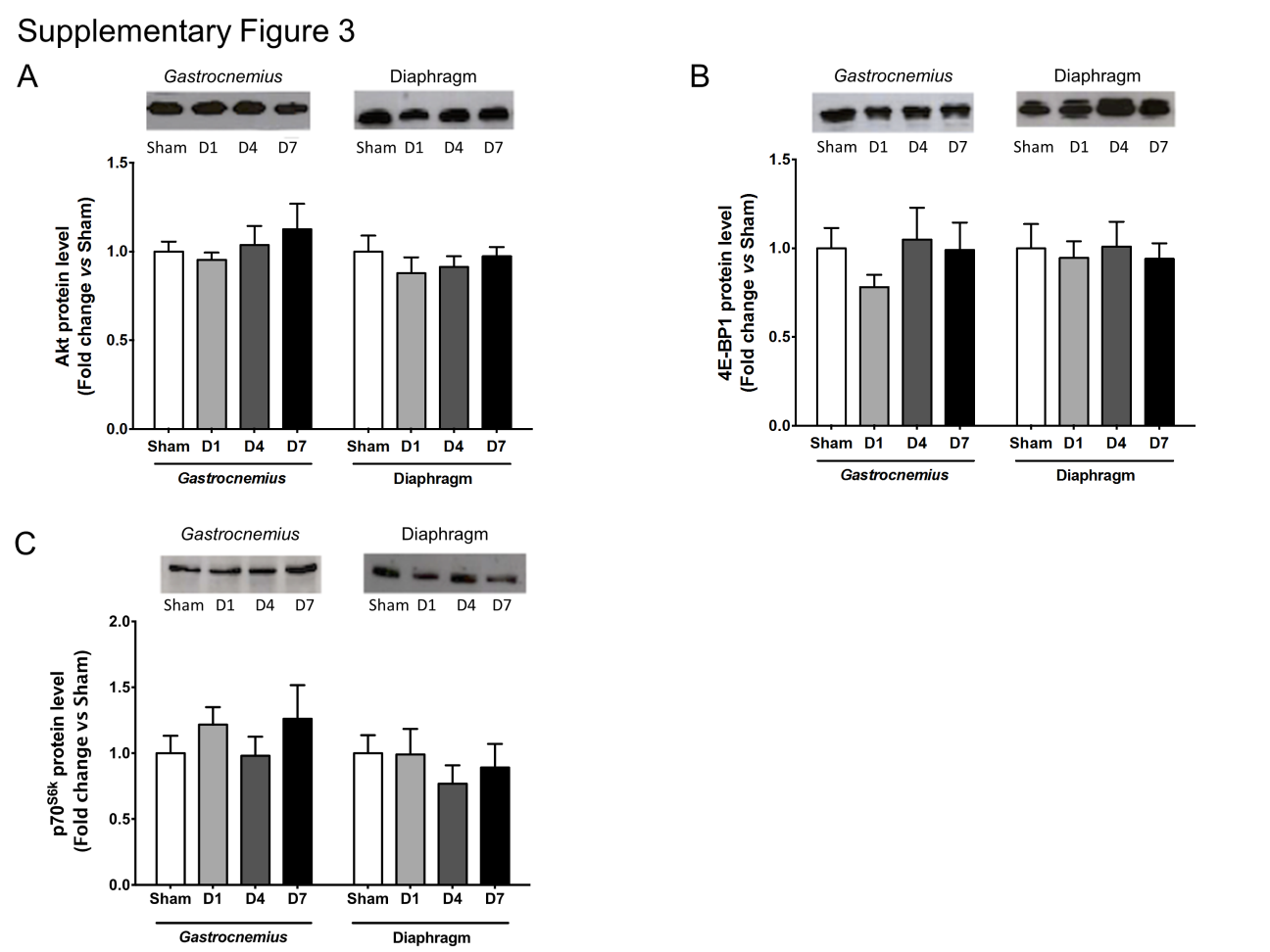


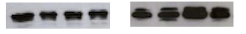


Diaphragm

Sham D1 D4 D7

**Supplementary Figure 4.** Comparative analysis of IL-1β, IL-6, TNF-α and IL-15 transcript levels in *gastrocnemius* (Gas) and diaphragm (Dia) muscles of sham animals. Data are expressed arbitrary units and presented as means ± SEM (n= 7-8/group). **, p < 0.01 and *** p < 0.001: significantly different from *gastrocnemius* muscle.

**SUPPLEMENTARY TABLE**

Supplemental Digital Content – Table 1: RNA forward and reverse primer sequences

| Gene | Primer sequence 5’-3’ | GenBank® accession no. |
| --- | --- | --- |
| α-tubulin | Fwd : TGA GGA GGT TGG TGT GGA TTC  Rev : Rev : AAA CAT CCC TGT GGA AGC AG | NM_011653.2 |
| Atg5 | Fwd : TGA AAG AGT GTG TCC TCC TC  Rev : GCC TCC ACT GAA CTT GAC TG | NM_053069.5 |
| ActRIIB (Acvr2B) | Fwd : ATC AGG AGG TCG GTC AAC  Rev : GAC TCT TTA GGG AGC AGG TC | NM_007397.2 |
| FoxO3 | Fwd : AGG ATA AGG GCG ACA GCA AC  Rev : CAT TCT GAA CGC GCA TGA AG | NM_019740.2 |
| Hprt | Fwd : CAG GCC AGA CTT TGT TGG AT  Rev : TTG CGC TCA TCT TAG GCT TT | NM_013556.2 |
| IL-1β | Fwd : GAA ATG CCA CCT TTT GAC AGT G  Rev : TGG ATG CTC TCA TCA GGA CAG | NM_008361 |
| IL-6 | Fwd : TAG TCC TTC CTA CCC CAA TTT CC  Rev : TTG GTC CTT AGC CAC TCC TTC | NM_031168.1 |
| IL-15 | Fwd : ACA TCC ATC TCG TGC TAC TTG T  Rev : GCC TCT GTT TTA GGG AGA CCT | NM_008357.2 |
| LC3b | Fwd : CAC TGC TCT GTC TTG TGT AGG TTG  Rev : TCG TTG TGC CTT TAT TAG TGC ATC | NM_026160.4 |
| MAFbx/atrogin-1 (Fbxo32) | Fwd : GTT TTC AGC AGG CCA AGA AG  Rev : TTG CCA GAG AAC ACG CTA TG | NM_026346.3 |
| MuRF1 (Trim63) | Fwd : ACC TGC TGG TGG AAA ACA TC  Rev : AGG AGC AAG TAG GCA CCT CA | NM_001039048.2 |
| Musa1 | Fwd : TCG TGG AAT GGT AAT CTT GC  Rev : CCT CCC GTT TCT CTA TCA CG | NM_001168297.1 |
| Myostatin | Fwd : ACG TCC AGA GGG ATG ACA GCA G  Rev : ACA TTT GGG CTT GCC ATC CGC | NM_010834.2 |
| P0 (Rplp0) | Fwd : CTC CAA GCA GAT GCA GCA GA  Rev : ATA GCC TTG CGC ATC ATG GT | NM_007475.5 |
| TNF-α | Fwd : TCC CAG GTT CTC TTC AAG GGA  Rev : GGT GAG GAG CAC GTA GTC GG | NM_001278601.1 |
| Ulk1 | Fwd : TTC CTG TCA GTC TGG CTC CT  Rev : TGA ACA GAG CCG TGA CAA AG | NM_009469.3 |
